# Application of Artificial Intelligence to an Impact-Based Analysis Method to Estimate the Soft Tissue Phantom Stiffness: A Proof-of-Concept

**DOI:** 10.3390/s26113594

**Published:** 2026-06-05

**Authors:** Arthur Bouffandeau, Felipe Rocha, Patrick Karasinski, Vu-Hieu Nguyen, Romain Bosc, Guillaume Haïat

**Affiliations:** 1 Laboratoire Modélisation et Simulation Multi Echelle, Centre National de la Recherche Scientifique, MSME UMR 8208 CNRS, 61 Avenue du Général de Gaulle, 94010 Creteil, France; 2 Laboratoire Modélisation et Simulation Multi Echelle, Université Paris Est Créteil, MSME UMR 8208 CNRS, 61 Avenue du Général de Gaulle, 94010 Creteil, France; 3 Service de Chirurgie Plastique, Reconstructrice, Esthétique et Maxillo-Faciale, Hôpital Henri Mondor APHP, Laboratoire IMRB INSERM U955—Université Paris—Est Créteil, 8 rue du Général Sarrail, 94010 Creteil, France

**Keywords:** soft tissues, impact analysis, elastic properties identification, machine learning, convolutional neural network, dermatology, cosmetics

## Abstract

Assessing the biomechanical properties of soft tissues can be useful because they are related to their pathophysiological state. This study explores the application of artificial intelligence (AI) to an Impact-Based Analysis Method (IBAM) to predict the mechanical properties of soft tissues. 40 agar-based soft tissue phantoms with different stiffness were prepared. For each phantom, Young’s modulus was estimated using dynamic mechanical analysis and IBAM measurements were performed. Various AI-based models were applied to the results obtained with the IBAM approach to predict Young’s modulus. Principal component analysis shows that three parameters can explain 85% of the variation in IBAM data. The times of the different maxima of the force signal peaks are significantly correlated with Young’s modulus ($R^{2}$ = 0.99). Most AI-based models allow a decrease in the prediction error compared to the standard IBAM approach (from 5.4% down to 1.2%). Decision trees and ensemble stacking, as well as convolutional neural networks, show a decrease in the prediction error of 74% and 50%, respectively. Applying AI approaches within the IBAM framework is a powerful approach to identify Young’s modulus of soft tissues. This study paves the way for using AI-based methods to characterize superficial soft tissue biomechanical properties.

## 1. Introduction

The mechanical properties of soft tissues are strongly dependent on their physiopathological conditions [1,2,3]. The assessments of these properties are essential both to understand basic biological phenomena and for the diagnosis of various diseases. Therefore, different elastographic techniques have been introduced over the last three decades to assess the biomechanical properties of deeply located soft tissues [4,5,6]. Today, elastographic techniques can be used to diagnose liver fibrosis [7,8], breast [9] or prostate cancer [5]. However, elastography is costly, relatively complex and difficult to apply for superficial soft tissues, such as skin [10,11]. Elastography remains difficult to use for superficial tissue assessment. Therefore, other methods, which should be user-friendly, low-cost, real-time, non-invasive and objective, should be developed to characterize the mechanical properties of superficial soft tissues, which could be highly promising and lead to valuable clinical applications. Note that measuring deep organs lies outside of the intended scope of IBAM, which is designed exclusively for superficial tissues. For instance, the Cutometer^®^ is a standard device in the cosmetics sector, based on the principle of suction to measure the viscoelastic behavior of the skin [12,13,14]. MyotonPro^®^ is a device designed to assess the biomechanical properties of soft tissues through the analysis of their response to a mechanical impulse [15]. It is used exclusively for research in physiotherapy, sports medicine [15,16] and dermatology [17,18].

A technique for the mechanical characterization of superficial soft tissues, named the Impact-Based Analysis Method (IBAM), has recently been developed by our group. It derives from applications related to total hip replacement surgery [19,20,21,22] and osteotomy procedures [23,24,25,26,27]. The advantage of IBAM is to be a simple, real-time, low-cost and non-invasive superficial soft tissue measurement device. This approach could become a valuable decision support tool in many medical fields, including dermatology and plastic surgery. Like the Cutometer^®^, it could be of great interest to the cosmetics industry. The principle is based on the measurement of the force signal produced when a hammer equipped with a force sensor impacts a punch in contact with the soft tissue to be characterized [28]. First, *in vitro* studies were carried out with IBAM and studied the performances of the technique (reproducibility, sensitivity and resolution). These studies showed that the time difference between the first two peaks (denoted Δt) was related to the stiffness of soft tissue mimicking phantoms [28,29]. These initial results showed that the stiffness sensitivity was equivalent to that of dynamic mechanical analysis (DMA), and was at least twice better than elastographic techniques such as magnetic resonance elastography (MRE) or optical coherence elastography (OCE). Then, another *in vitro* study compared the performances of IBAM with other devices such as Cutometer^®^, MyotonPro^®^, IndentoPro^®^ and Shore Durometer [30]. IBAM was shown to be the most effective mechanical characterization approach for superficial soft tissue in terms of stiffness and spatial sensitivity. Second, modeling work was carried out to improve the understanding of the physical phenomena occurring during an IBAM measurement, using a 1D analytical model and a finite element (FE) model [31]. Third, preliminary *ex vivo* and *in vivo* experiments were conducted with murine skin models. Cutaneous neurofibromas could be differentiated from healthy regions on the basis of their mechanical behavior using IBAM [32]. In comparison with Cutometer^®^ and MyotonPro^®^, IBAM demonstrated its aptitude for monitoring *in vivo* variations in the mechanical properties of superficial soft tissues of rats during wound healing or after a hyaluronic acid injection [33].

Considering the number of factors affecting IBAM measurements, machine learning (ML) approaches may be interesting solutions to improve the performance of the method due to their capacity for expressiveness without restrictive assumptions [34]. ML is a subfield of artificial intelligence (AI) that uses mathematical and statistical methods to identify nonlinear, multidimensional patterns or relationships from training datasets. Unsupervised learning methods (e.g., clustering techniques such as K-means or uniform manifold approximation and projection, UMAP) aim to uncover established relationships or recognize structures from unlabeled data, whereas supervised learning methods (e.g., artificial neural networks (ANN), decision trees, k-nearest neighbors (KNN), and support vector machines (SVM)) rely on labeled data to classify or predict outcomes with known inputs and outputs. Reinforcement learning is a type of ML in which models interact with their environment iteratively and use a reward system to accumulate training. Among these various approaches, the models presented in this paper will be based exclusively on supervised learning. Deep learning (DL) is a subfield of ANN with a high number of neurons and layers that is adapted to derive new representations of large datasets. Contrary to classical ML approaches, these methods do not require any prior modification of the input data, such as the extraction of signal characteristics. Convolutional neural networks (CNNs) are one of the most common structures for image-based DL applications [35]. CNNs are currently widely used for image classification tasks such as histological image processing and cancer diagnosis due to their excellent performance. Various ML methods have been applied to estimate the mechanical properties of different materials, such as metals, polymers, and composites [34]. These approaches include ANN [36] and decision trees [37]. Different studies have investigated the implementation of ML approaches to assess the material parameters of biomaterials and biological soft tissues [38]. For instance, a Gaussian process regression model was used to predict skin pre-stretch from surface wave speed [39]. However, the model training and testing data (i.e., stressing states and surface wave propagations) were generated from a FE model of the skin [39]. For impact analysis applications to osteotomy procedures, AI-based approaches using SVM classifiers were tested with excellent results. An *in vitro* study used SVM to classify different materials according to the mechanical properties (stiffness and thickness) using impact analysis data (89% accuracy) [23]. Then, similar models were used to detect bone crossing in *ex vivo* measurements with animal samples during pterygomaxillary disjunction (95% accuracy) [27] and *in vivo* on human cadavers during rhinoplasty (83% accuracy) [24]. However, AI-based approaches have never been applied to IBAM measurements in the context of soft tissue assessment.

This paper explores the potential applications of AI-based methods to analyze the data obtained with the application of IBAM to superficial soft tissues. The aim is to determine whether AI-based approaches could be useful to improve IBAM performances. Firstly, a collection of agar-based soft tissue phantoms manufactured by varying agar mass concentration by 0.1% was realized. Secondly, IBAM measurements and Young’s modulus estimation were performed for each sample. Thirdly, principal component analysis (PCA) was conducted on these data to explore their structure and identify potential relationships. Fourthly, prediction models of Young’s modulus from IBAM force signals were implemented by means of various regression ML methods such as decision trees, SVM and CNN. The prediction error of these different models was then compared with the IBAM reference error, which was calculated using the indicator Δt.

## 2. Materials and Methods

### 2.1. Soft Tissues Phantoms Preparation

The database of soft tissue phantoms for model training was constituted by a large collection of agar hydrogel samples, realized with 40 values of agar mass concentrations $c_{ag}$ ranging from 1.5% to 5.4% with a 0.1% step. Following the protocol described in Bouffandeau et al. [29], a hydrogel of concentration $c_{ag}$ was prepared by diluting a mass $m_{ag}$ of agar powder in a volume $V_{w}$ of distilled water, such as:

(1) $$m_{ag}=\frac{c_{ag}\;\rho_{w}\;V_{w}}{1-c_{ag}},$$

where $\rho_{w}$ corresponds to the density of the distilled water. The solution was mixed using a heating magnetic stirrer, heated to boiling point and then cooled to 60 °C by continuing stirring. At this target temperature, the mixture was poured into a cylindrical mold (diameter ⌀ = 20 mm and length L = 45 mm) and the gelation process was finalized for 20 min in the refrigerator at 5 °C. The agar hydrogel sample was then removed from its mold and immersed in distilled water in the refrigerator. It was stored for at least one night (minimum 12 h). One hour prior to manipulation, the sample was taken out of the refrigerator and transferred to fresh distilled water to reach room temperature (approximately 19 °C). It was removed from the water just before the measurements. Because of their predominant water composition, the agar hydrogel samples were kept in water to prevent dehydration and their exposure to ambient air was limited to 10 min [40].

### 2.2. Mechanical Characterization

Each agar hydrogel sample was mechanically characterized using two approaches: i) DMA via a custom vibration set-up (to estimate the reference value of dynamic Young’s modulus $E_{ref}$) and ii) IBAM, as described below. Subsequently, the results obtained were compared.

#### 2.2.1. Young’s Modulus Estimation

**Figure 1:**
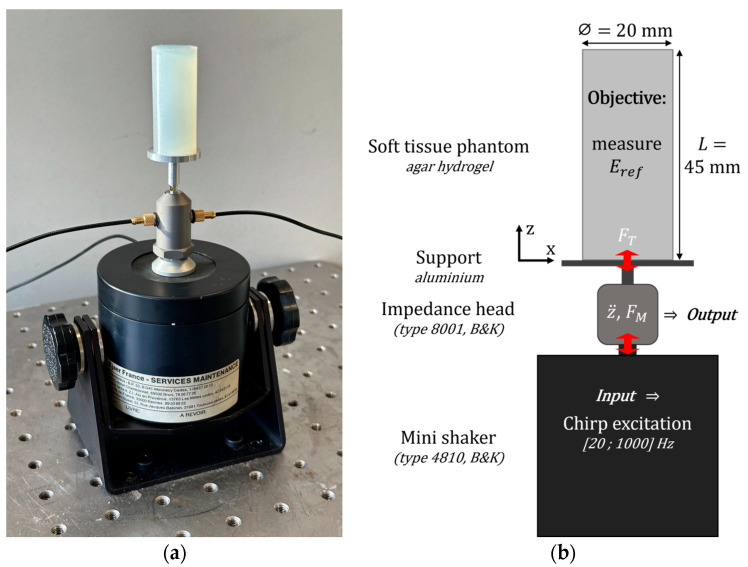
(**a**) Custom vibration set-up used to estimate Young’s modulus $E_{ref}$ of soft tissue phantoms. (**b**) Illustration of the principle of $E_{ref}$ estimation. The shaker emits a chirp mechanical displacement excitation. The impedance head records the force and acceleration response of the system {phantom + support}, $F_{M}\left(t\right)$ and $\ddot{z}\left(t\right)$ respectively. Assuming the sample is an elastic beam, $E_{ref}$ is determined from the fundamental resonance frequency $f_{1}$ of the corrected transfer function $Y_{C}\left(f\right)$, obtained from $F_{M}\left(t\right)$ and $\ddot{z}\left(t\right)$.

Similarly to previous experiments [29], a custom vibration set-up was used to assess $E_{ref}$. Briefly, the principle consisted in interpreting the vibrational response of soft tissue phantoms to mechanical excitation in order to derive their mechanical properties under dynamic states, assuming an isotropic linear elastic behavior. The viscous phenomena of soft tissue phantoms were neglected. The sample was positioned on an aluminum support connected to an impedance head (type 8001, Brüel and Kjær (B&K), Naerum, Denmark) coupled to a mini shaker (type 4810, B&K, Naerum, Denmark), as illustrated in Figure 1. The mini shaker provided the mechanical displacement excitation as a chirp function between 20 and 1000 Hz, with peak-to-peak amplitudes of around 0.03 mm. The impedance head was used to record the response of the system {phantom + support} in terms of vertical force $F_{M}\left(t\right)$ and vertical acceleration $\ddot{z}\left(t\right)$, according to the z-axis. By modeling the system as a linear elastic beam (⌀ = 20 mm & L = 45 mm), the vibrational response of the phantom could be expressed as a function of a single spatial coordinate, which corresponds to the longitudinal axis of the beam, using the longitudinal vibration theory of beams [41]. The dimensions of the phantoms (L > 2 ⌀) fulfilled the two necessary conditions of the theory: i) the cross-section remains planar during deformation, and ii) all displacement components are negligible except for those parallel to the longitudinal axis. Thus, in the case where the beam was free at its upper extremity (at z = L) and a displacement was imposed at its lower extremity (at z = 0 mm), the dynamic Young’s modulus $E_{ref}$ could be determined using the fundamental resonance frequency $f_{1}$ of the transfer function of the Fourier transforms of acceleration on force following:

(2) $$E_{ref}=4\;\rho \;{\left(L\;f_{1}\right)}^{2},$$

Due to the dynamic nature of the oscillation, the material response may differ from the static behavior, which can lead to an overestimation of the value and frequency dependence of the measured dynamic Young’s modulus compared to the static Young’s modulus.

Considering the positioning of the support between the sample and the impedance head, and its non-negligible mass compared to that of the sample, the force measured $F_{M}\left(t\right)$ must be corrected [42,43]. At the impedance head, the measured force $F_{M}\left(t\right)$ was the sum of the force transmitted to the sample $F_{T}\left(t\right)$ and the force applied to the support of the sample $F_{S}\left(t\right)$, as follows:

(3) $$F_{M}\left(t\right)=F_{T}\left(t\right)+F_{S}\left(t\right),$$

Then, from the Fourier transforms of the different variables, the measured and corrected transfer functions, $Y_{M}\left(f\right)$ and $Y_{C}\left(f\right)$ respectively, could be defined as:

(4) $$Y_{M}\left(f\right)=\frac{\hat{\ddot{z}}\left(f\right)}{{\hat{F}}_{M}\left(f\right)}\;\mathrm{and}\;Y_{C}\left(f\right)=\frac{\hat{\ddot{z}}\left(f\right)}{{\hat{F}}_{T}\left(f\right)},$$

where ${\hat{F}}_{M}\left(f\right)$, ${\hat{F}}_{T}\left(f\right)$, ${\hat{F}}_{S}\left(f\right)$ and $\hat{\ddot{z}}\left(f\right)$ corresponded to the Fourier transforms of $F_{M}\left(t\right)$, $F_{T}\left(t\right)$, $F_{S}\left(t\right)$ and $\ddot{z}\left(t\right)$. Nevertheless, to express ${\hat{F}}_{S}\left(f\right)$, a measurement had to be performed without a sample. In a no-load system, the measured force $F_{M_{nl}}\left(t\right)$ and acceleration ${\ddot{z}}_{nl}\left(t\right)$ values (respectively, their Fourier transforms ${\hat{F}}_{M_{nl}}\left(f\right)$ and ${\hat{\ddot{z}}}_{nl}\left(f\right)$) were recorded and used to estimate the apparent spectral mass $m_{S}\left(f\right)$ [44,45] such as:

(5) $$m_{S}\left(f\right)=\frac{{\hat{F}}_{M_{nl}}\left(f\right)}{{\hat{\ddot{z}}}_{nl}\left(f\right)},$$

(6) $${\hat{F}}_{S}\left(f\right)=m_{S}\left(f\right)\times \hat{\ddot{z}}\left(f\right),$$

Lastly, also following [44,45], the corrected transfer function $Y_{C}\left(f\right)$ could be expressed in terms of the impedance head, output variables, and the apparent spectral mass $m_{S}\left(f\right)$ as follows.

(7) $$Y_{C}\left(f\right)=\frac{\hat{\ddot{z}}\left(f\right)}{{\hat{F}}_{T}\left(f\right)}=\frac{\hat{\ddot{z}}\left(f\right)}{{\hat{F}}_{M}\left(f\right)-m_{S}\left(f\right)\times \hat{\ddot{z}}\left(f\right)}=\frac{Y_{M}\left(f\right)}{1-m_{S}\left(f\right)\times Y_{M}\left(f\right)},$$

Finally, after this mass correction, $f_{1}$ corresponded to the fundamental resonance frequency of $Y_{C}\left(f\right)$. For each sample, the measurement was repeated three times and the value of $E_{ref}$ corresponded to the average of the three measured values.

#### 2.2.2. Impact-Based Analysis Method (IBAM)

**Figure 2:**
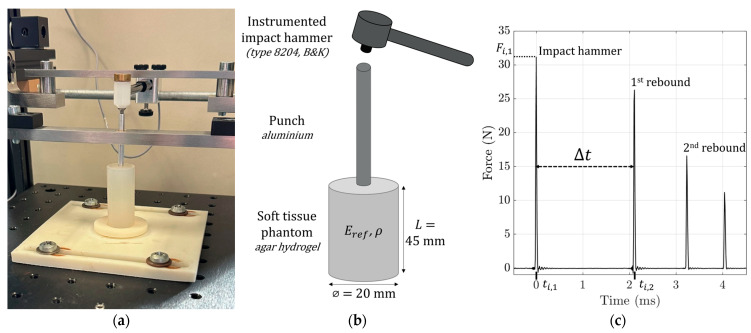
(**a**) Experimental set-up of the impact-based analysis method (IBAM). (**b**) Schematic illustration of the experimental set-up of IBAM. (**c**) Typical shape of a force signal with the significance of the Δt indicator. In this instance, $F_{1}$ = 31.19 N and Δt = 2.11 ms.

The experimental set-up of IBAM was described in Bouffandeau et al. [30] and is briefly recalled herein. Figure 2a illustrates the two main components of the system: an impact hammer instrumented with a force transducer (type 8204, B&K, Naerum, Denmark) and a cylindrical punch with a diameter of 4 mm. A measurement resulted from the impact of the hammer on the punch, whose lower extremity was in contact with the soft tissue to be characterized. The force sensor of the hammer recorded the force signal with a sampling frequency of 102.4 kHz (NI 9232, National Instruments, Austin, TX, USA) using LabVIEW (National Instruments, Austin, TX, USA). The amplitude of the first peak $F_{1}$ and the positions of the first two peaks $t_{1}$ and $t_{2}$ were determined. The force peaks coincided with the hammer impact on the punch and the initial rebounds of the punch on the hammer [28], as shown in Figure 2b. The indicators Δt were determined for signals with $F_{1}$ ∈ [30; 35] N following:

(8) $$\Delta t=t_{2}-t_{1},$$

For each soft tissue phantom, a series of measurements was composed of 25 distinct Δt measurements, corresponding to 25 impacts [29]. Three series of measurements were performed by repositioning the sample and the punch. Furthermore, for each series of measurements, an outlier detection method was implemented based on the z-score metric, with a threshold of 2 for a 95% confidence interval [46,47]. Additionally, particular attention was paid to punch guidance to minimize friction, which could lead to a degradation of the repeatability of the force signal and increase damping of the system, interfering with the estimation of the intrinsic viscosity of soft tissue. This improvement was achieved by using a self-lubricating guide ring and was also achieved by ensuring a sliding fit between the punch and the guide ring.

#### 2.2.3. Database

For the regression models described below, the datasets used were composed of the data obtained with IBAM. It is composed of i) 3000 signals corresponding to 3 repetitions of 25 impacts for each agar hydrogel sample and ii) the estimated values of Young’s modulus $E_{ref}$ (40 values, one for each sample) of soft tissue phantoms. The data from IBAM corresponded to the features (input data of algorithms) and were used to predict the value of Young’s modulus, whereas $E_{ref}$ corresponded to the label. Depending on the ML and DL models, the nature and formatting of the features (IBAM data) may vary between the datasets.

**Figure 3:**
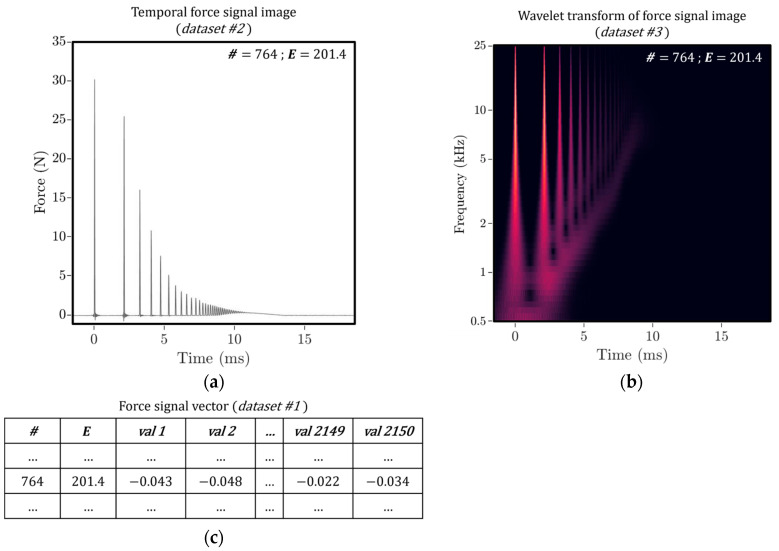

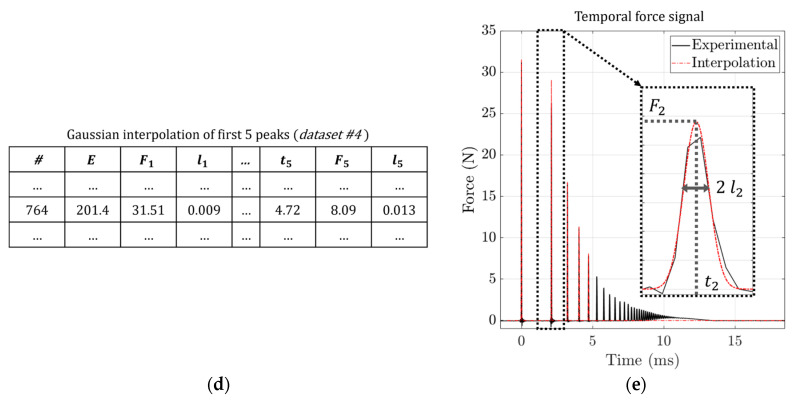
Example of features formatting from a force signal of IBAM in the various datasets. For all datasets, each signal is identified by a unique identifier # (764) and labeled with an $E_{ref}$ value (201.4 kPa). (**a**) *Dataset #2*: plot of force signal as a function of time. (**b**) *Dataset #3*: plot of the wavelet transform (horizontal axis: time domain and vertical axis: frequency domain). For both images, the plot parameters were constant between signals despite the absence of axes, scales and grids. (**c**) *Dataset #1*: amplitude vector of sampled force signal (native form). (**d**) *Dataset #4*: set of parameters of the first 5 peaks with $F_{i}$, $l_{i}$ and $t_{i}$ (respectively, the amplitude, half-width and position of the ith peaks determined by Gaussian interpolation). (**e**) Example of Gaussian interpolation for the second peak.

First, the nature of the feature was given by a force vector corresponding to the signal with 2150 samples and a constant sampling frequency of 102.4 kHz, as illustrated in Figure 3c. This dataset (numbered *#1*) was used with a 1D-CNN model, described in Section 2.4.4. Second, the feature was an image of the curve corresponding to the variation in the force as a function of time, as shown in Figure 3a, which was plotted using the Matplotlib module [48] (version 3.8.4) with Python [49] (version 3.10.13). This dataset (numbered *#2*) was then used in a 2D-CNN model, described in Section 2.4.4. Third, the nature of the feature was a wavelet transform applied to the force signal. An A wavelet transform was performed using the PyWavelet module [50] (version 1.6.0) with Python [49] (version 3.10.13). The result was represented as a scalogram of the force signal with frequency as a function of time (in the intervals [0; 20] ms, [500; 25000] Hz and [0; 10] N) using Matplotlib (version 3.8.4), as shown in Figure 3b. This dataset was numbered *#3*. As with the other datasets, the scalogram images were labeled with a unique identifier and their corresponding $E_{ref}$, for use in a 2D-CNN model, described in Section 2.4.4. Similarly to the other image, the axes, graduations and grids were removed, as shown in Figure 3b. Fourth, the nature of the feature was a set of parameters describing the signal peak characteristics, as shown in Figure 3d. These parameters were obtained by performing a Gaussian interpolation of the first five peaks of the signal, as illustrated in Figure 3e. For the peak *#*i (i ∈ [1; 5]), the time of the maximum $t_{i}$, the amplitude $F_{i}$ and the half-width $l_{i}$ of the Gaussian interpolation were determined. Note that the time origin was defined as the time of the first peak, so that $t_{1}$ = 0 ms. This dataset was numbered *#4*. Similarly, each set of parameters was associated with a unique identifier and labeled with its corresponding $E_{ref}$. Then, these datasets were used in the data analysis and ML regression models, described in Section 2.3 and Section 2.4.3.

### 2.3. Data Analysis

A data analysis was performed using the dataset of interpolated peak parameters (*dataset #4* with 3000 samples, 14 features and 1 label) to identify any relationship between these parameters and the stiffness phantoms. First, the linear regression analysis was performed for each set of parameters $t_{i}$, $F_{i}$ and $l_{i}$, two by two, and the correlation coefficients were summarized in a correlation matrix. Second, PCA was carried out on this same dataset to reduce its dimensionality, while capturing the maximum variance of this dataset. To do so, the sample values were expressed using new and uncorrelated variables, which were linear combinations of the intercorrelated original features [51]. By associating the data with their label $E_{ref}$ and projecting them into the reduced space, it was possible to identify underlying structures between the features and labels of the dataset. The principal components were selected using the cumulative percentage of total variance approach [51]. The principal components with the greatest reported variance were selected one by one until their cumulative variance exceeded 80%.

### 2.4. Young’s Modulus Prediction

#### 2.4.1. IBAM

Firstly, a power law regression analysis was performed for the values of Δt as a function $E_{ref}$. Then, by reformulating the fit relation, the predicted Young’s modulus value $E_{pred}$ could be expressed from Δt. Finally, the prediction error of Δt was quantified from the root mean squared percent error RMSPE such as:

(9) $${RMSPE}_{\Delta t}=\sqrt{\frac{1}{n}\sum_{i=1}^{n}{\left(\frac{E_{pred}-E_{ref}}{E_{ref}}\right)}^{2}},$$

where $E_{pred}$ and $E_{ref}$ were the predicted and reference Young’s modulus values, respectively, and n was the number of samples in the dataset. The performance of the various regression models described below was also evaluated using the metric RMSPE.

#### 2.4.2. Predictive Artificial Intelligence

Two types of regression models could be distinguished, i) classical ML models and ii) DL models, which were designed around CNN. Supervised learning was employed for all these models, with the datasets split randomly into two distinct subsets: 85% of the data for training and validation, and the remaining 15% for final performance testing. The cross-validation method was incorporated into the training process by dividing the training data into five subsets. This approach increased the training volume artificially and reduced the bias and variability present in the training data. After optimizing their parameters and selecting their hyperparameters during training, each regression model predicted Young’s modulus $E_{pred}$ using the subset of data reserved for final testing. The prediction error was quantified using the RMSPE metric, as defined in Equation (9), and then compared with the reference value ${RMSPE}_{\Delta t}$.

#### 2.4.3. Machine Learning

Various ML models were compared to predict Young’s modulus of soft tissue phantoms based on the 14 characteristic peak parameters derived from the IBAM signal (see Section 2.2.3). First, the simplest approach was linear regression analysis and consisted of identifying the linear combination of variables that minimized the sum of the squares of differences between $E_{ref}$ and the predicted $E_{pred}$ values [52]. Second, the KNN regression algorithm was also used, as the distribution of label values $E_{ref}$ were smooth. Here, the prediction values $E_{pred}$ of a new sample was obtained by taking the weighted average of the labels $E_{ref}$ associated with the k nearest-neighbor in the samples [52]. Third, an SVM model was considered. Compared to classification approaches, the purpose was not to maximize the separation of the data with a hyperplane, but to identify the subspace that best approximates the behavior of the dataset [52]. Fourth, a stochastic model was applied using a Gaussian process regression to retrieve the distribution generating the training data under the assumption of Gaussian data distributions [53]. Decision trees, which correspond to a decision-making diagram, were also implemented through different versions of these models, such as Gradient Boosting algorithms (e.g., CatBoost and XGBoost) and Random Forest algorithm. The algorithms of Gradient Boosting consisted of successively combining decision trees, with each one correcting the weaknesses of the previous ones. The algorithms of Random Forest were sets of independent decision trees with an output based on a synthesis process (e.g., average or majority). From the previously described approaches, the best regression models were combined in a stacking ensemble model to provide the optimal prediction [52,54,55].

The Grid Search method was used in the training process to optimize hyperparameters, detailed in Table A1. Moreover, a model inspection technique based on the permutation feature importance method was used to evaluate the influence of each variable on the prediction of the various final models [56].

#### 2.4.4. Deep Learning

A supervised DL approach was developed using a CNN from the Keras library [57] (version 3.4.1) on a Python environment (version 3.10.13). Three different approaches were developed with one-dimensional (1D-CNN) and two-dimensional (2D-CNN) input data, as described in Section 2.2.3. The 1D-CNN was developed from one-dimensional input data, such as vectors of IBAM force signals, as shown in Figure 3c. The 2D-CNNs were developed from two-dimensional input data, such as graphs of force signals as a function of time or scalograms resulting from the wavelet transform of IBAM force signals, as shown in Figure 3a and Figure 3b, respectively.

**Figure 4:**
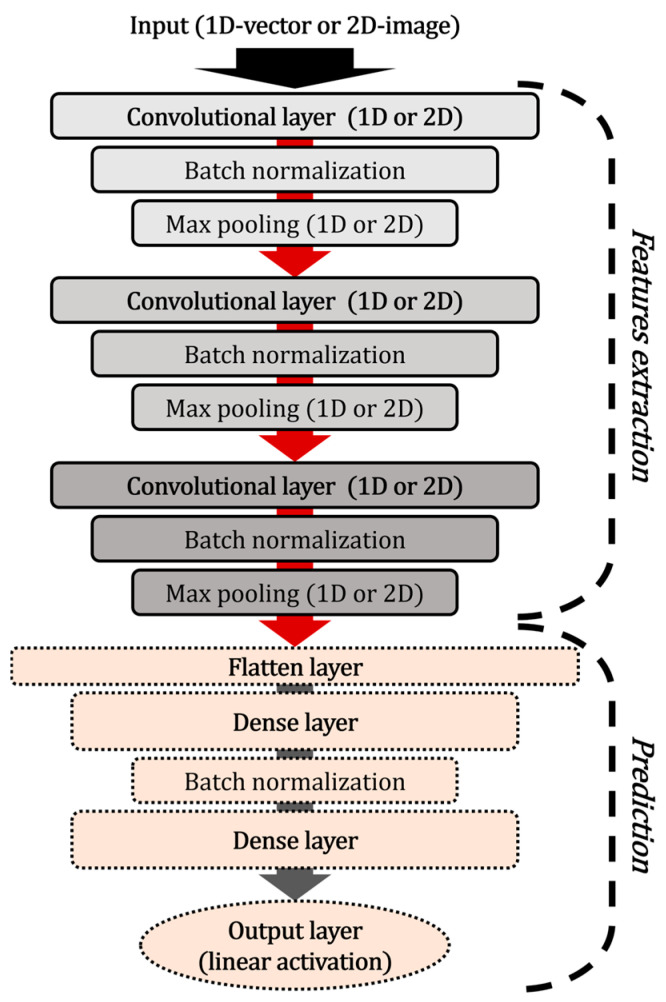
Structure of CNNs: the first three layer groups for feature extraction and the final layers for synthesizing and predicting a Young’s modulus value, $E_{pred}$. The convolution layers are adapted to input dimensions of one and two, respectively, for force signal vectors and images of temporal force signals and scalograms induced by wavelet transforms of force signals.

The CNN architecture included a succession of multiple layers of neurons, whose first layers were used to extract features from the input data. The last layers were used to compile the results and predict the output, as shown in Figure 4. The result of each operation or filter from one layer constituted the input for the next layer [58]. Three types of layers composing the feature extraction were distinguished. First, depending on the form of the input data (vectors or images), convolution layers (1D or 2D) applied convolution filters to detect characteristics of the data, ranging from simple for the first layers (e.g., contours and textures) to highly abstract elements for the latter. The layer was enhanced by activation functions, such as the rectified linear unit (ReLU) function, to promote the transmission of certain characteristics between layers. Padding techniques were also used at borders to preserve the dimensionality of data during filtering. Second, pooling layers were used between the convolution layers to reduce the dimensions of the data using a subsampling filter. For instance, the max pooling method transmitted the most important value from the previous layer to the next layer. Third, regularization techniques (e.g., batch normalization function) were added after each pooling layer to accelerate model training and prevent overfitting.

After the last feature extraction layer, several layers predicted the output value from the large amount of data generated by the previous phase. Thus, this phase was composed only of dense layers. These layers were also supplemented by ReLU activation functions, except for the output layer, which had a linear activation function to enable regression. However, if the dimension of the final layer of the feature extraction phase was superior to 1, a flatten function was applied, prior to the first dense layers, in order to obtain a one-dimensional tensor. For these CNNs, parameter tuning was carried out using the Adam optimizer with a learning rate of 0.001 and RMSPE as loss function, defined in Equation (9). The batch size was specified to 32 for training, with a maximum of 500 epochs. Two callback functions (checkpoint model and early stop) were also integrated into the training process.

## 3. Results

### 3.1. Young’s Modulus Estimation

**Figure 5:**
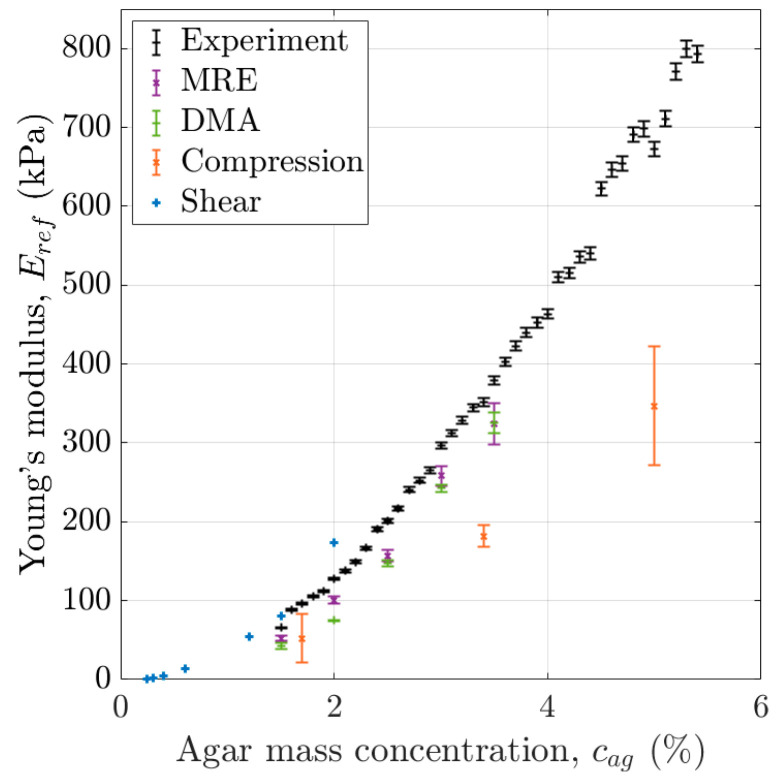
Comparison of the variation in Young’s modulus $E_{ref}$ of agar hydrogel samples as a function of agar mass concentration $c_{ag}$ using our mechanical characterization (Experiment), magnetic resonance elastography (MRE) [59], dynamic mechanical analysis (DMA) [59], quasi-static compression test (Compression) [60] and rheological tests (Shear) [61]. The error bars reflect the measurement reproducibility.

Figure 5 shows the variation in Young’s modulus $E_{ref}$ of agar hydrogel samples as a function of their mass concentration $c_{ag}$ ranging from 1.5% to 5.4% with a 0.1% step. The average difference in Young’s modulus between two consecutive samples was 18.7 ± 15.7 kPa. Notably, the values $E_{ref}$ are uniformly distributed over the interval of $c_{ag}$ ∈ [1.5%; 4%].

### 3.2. Data Analysis

**Figure 6:**
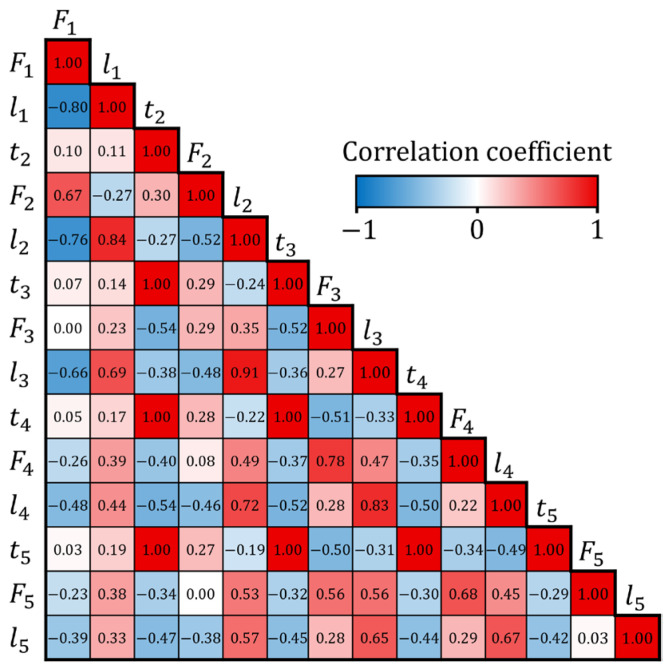
Correlation matrix of interpolated parameters of the first 5 peaks of force signals ($F_{i}$: amplitude, $l_{i}$: half-width and $t_{i}$: position of ith peaks).

Figure 6 shows the correlation matrix of the characteristics for the first five peaks of force signals for the entire dataset. A perfect positive correlation is observed between all $t_{i}$ and follow the linear relationships: $t_{3}$ = 1.521 × $t_{2}$, $t_{4}$ = 1.248 × $t_{3}$ and $t_{5}$ = 1.151 × $t_{4}$. The parameters $F_{1}$ and $l_{1}$ are highly negatively correlated. $F_{1}$ is also negatively correlated with $l_{2}$ and $l_{3}$. Positive correlations of more or less significance are also found between $l_{i}$ or $F_{i}$, the highest correlations occurring between neighboring peaks. For example, $F_{4}$ shows the highest correlation with $F_{3}$ and $F_{5}$.

**Figure 7:**
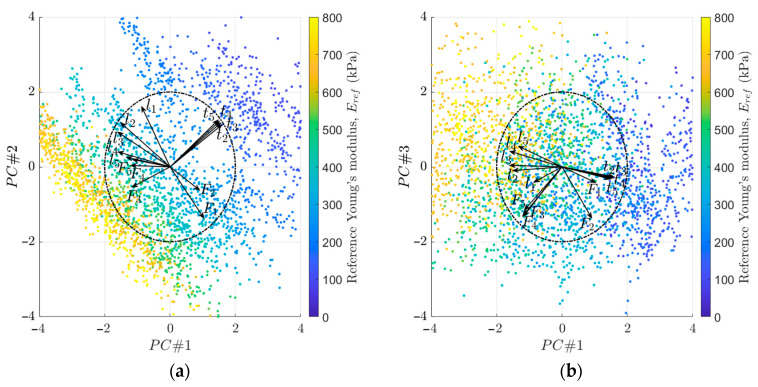
Projection of data labeled with $E_{ref}$ obtained by PCA and correlation circle of parameters $t_{i}$, $F_{i}$ and $l_{i}$ as function of the principal components with (**a**) *PC #1* and *PC #2*, and (**b**) *PC #1* and *PC #3*.

Figure 7 shows the projection of dataset values labeled with $E_{ref}$ within the space resulting from the PCA. The reduction analysis is restricted to three components capturing 85.2% of the dataset variance, which corresponds to 46.8% for the first principal component (*PC #1*), 24.2% for the second (*PC #2*) and 14.2% for the third (*PC #3*). As mentioned above, the various correlations existing between the parameters can explain this strong dimensionality reduction. The dataset values are colored with respect to their label $E_{ref}$ and plotted as a function of their coordinates *PC #1* and *PC #2* for Figure 7a, and *PC #1* and *PC #3* for Figure 7b in the new reduced space. Figure 7a shows a gradient of $E_{ref}$ in the plane of *PC #1* and *PC #2*. Therefore, the value of $E_{ref}$ for each dataset value can be predicted by a certain combination of *PC #1* and *PC #2* (which results themselves from a linear combination of the dataset features ($t_{i}$, $F_{i}$ and $l_{i}$)). In addition, the correlation circles identify the correlations between the dataset features ($t_{i}$, $F_{i}$ and $l_{i}$) and the principal components (*PC #1*, *PC #2* and *PC #3*). The parameters $t_{i}$ are strongly correlated with a combination of *PC #1* and *PC #2*. Furthermore, this alignment coincides with the gradient of $E_{ref}$, as shown in Figure 7a. Thus, IBAM is sensitive to Young’s modulus of the samples, particularly through the parameters $t_{i}$. The other dataset features ($F_{i}$ and $l_{i}$) do not align in specific directions or in accordance with the gradient of $E_{ref}$. However, in the first two peaks, the amplitudes $F_{1}$ and $F_{2}$ and the half-widths $l_{1}$ and $l_{2}$ are negatively correlated to each other, which is consistent with the peak shape.

### 3.3. Young’s Modulus Prediction

**Figure 8:**
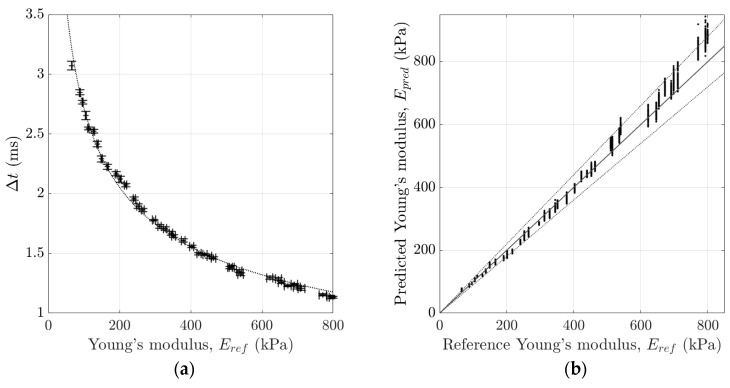
(**a**) Variation of Δt as a function of Young’s modulus $E_{ref}$. The error bars correspond to the reproducibility of the measurements. The dotted line corresponds to the power-law regression analysis (see Equation (10)). (**b**) Comparison of predicted value $E_{pred}$, using Δt values of IBAM, regarding the reference value $E_{ref}$ of Young’s modulus with deviations of ±10%.

Figure 8a shows the variation of Δt as a function of $E_{ref}$. The error bars represent the reproducibility of the measurements for Δt and $E_{ref}$. Δt decreases as a function of $E_{ref}$. A power-law regression analysis is performed, leading to:

(10) $$\Delta t=17.209\;{E_{ref}}^{-0.401}\;\mathrm{with}:\;R^{2}\;=\;0.99,$$

Equation (10) is then used to predict Young’s modulus $E_{pred}$ of phantoms from Δt values. Figure 8b shows the predictions of the IBAM model on Young’s modulus $E_{pred}$ as a function of reference values $E_{ref}$ with dotted lines indicating deviations of ±10%. The prediction error of this simple model is quantified by determining the RMSPE, which is equal to 5.84%.

Table 1 compares the performances (RMSPE) of the various models used to predict Young’s modulus of soft tissue phantoms from IBAM measurements. Except for the linear model with least squares regression, all the ML algorithms show a reduction of RMSPE compared to the prediction method using the Δt indicator. The performance of the linear regression model could be expected, since a raw linear combination of force signal peak characteristics struggles to capture nonlinear behavior, as shown in Figure 8a, and can lead to overfitting. Decision trees (CatBoost, XGBoost and Random Forest) and the ensemble stacking approach show the lowest prediction errors, with a RMSPE around 1 to 2%. The DL models (1D-CNN and 2D-CNN) demonstrate slightly higher prediction errors around 2% and 3%, but are still better than with the Δt indicator.

**Figure 9:**
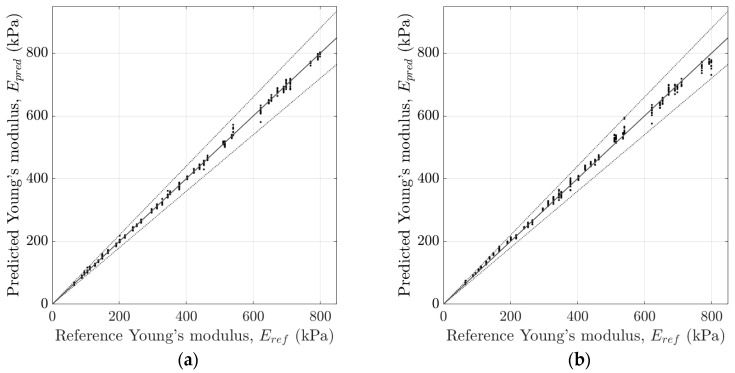
Comparison of predicted value $E_{pred}$ regarding the reference value $E_{ref}$ of Young’s modulus with deviations of ±10% using (**a**) decision tree with CatBoost algorithm or (**b**) 2D-CNN with images of force as function of time.

Figure 9 plots the predicted values $E_{pred}$ as a function of the reference values $E_{ref}$ of Young’s modulus with dotted lines indicating deviations of ±10% for the decision tree with Gradient Boosting, CatBoost, and the convolutional neural network (2D-CNN) using images of the temporal force signal from IBAM. Comparing the prediction results obtained with the indicator Δt, as shown in Figure 8b, both models show a decrease in scattering around the y = x axis, leading to a reduction in their RMSPE.

**Figure 10:**
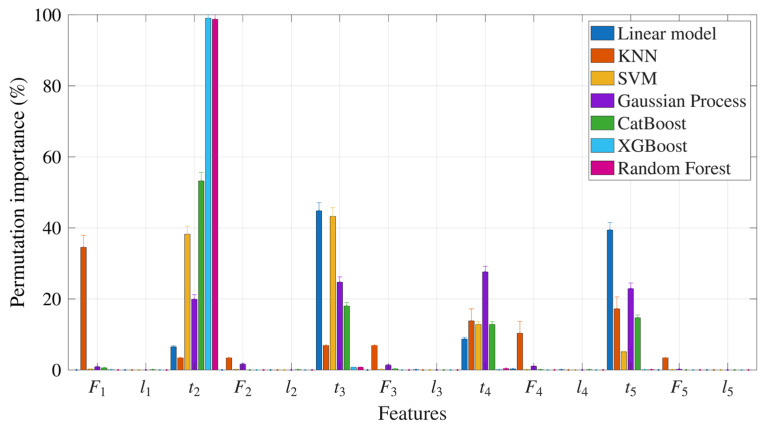
Comparison of feature influences on the prediction mechanisms of various machine learning models using permutation feature importance technique.

In the case of ML, the hyperparameters of various models are optimized using the Grid Search function, as explained in Section 2.4.3. In this way, the configuration with the lowest RMSPE is retained and specified in Table A1 for each model. Similarly, the stacking model is built from the optimal models and their associated hyperparameters: KNN, SVM, and the three decision trees (CatBoost, XGBoost and Random Forest). Figure 10 shows the results of the inspection technique to understand the prediction mechanisms of ML models using the permutation feature importance technique [56]. The permutation importance corresponds to the weight of the parameter in the prediction mechanism. For all models, the parameters $t_{i}$ are the most impactful features in the prediction, except for KNN, where $F_{1}$ is predominant. Inside the various decision trees, $t_{2}$ (i.e., indicator Δt) has the most important influence (permutation importance of over 50%), and even exclusive influence (permutation importance of almost 100%) for the XGBoost and Random Forest models. Thus, these last two models only rely on $t_{2}$ (i.e., indicator Δt) for their Young’s modulus prediction.

## 4. Discussion

The originality of this paper was to apply for the first time AI-based methods to the characterization of the biomechanical properties of superficial soft tissues. These developments could lead to better measurement methods of soft tissue biomechanical properties.

### 4.1. Soft Tissues Phantoms

Here, a collection of 40 agar hydrogel samples were prepared to mimic the mechanical behavior of soft tissues [62]. The objective was to obtain a nearly continuous variation in the soft tissues phantoms stiffness by varying the agar mass concentration $c_{ag}$ by 0.1%. Despite the significant variability of agar hydrogels with respect to fabrication and storage conditions [63,64], the dynamic Young’s modulus $E_{ref}$ of different phantoms increased uniformly as a function of $c_{ag}$, as illustrated in Figure 5. The stiffness range observed for these phantoms qualitatively matches literature data, where Young’s modulus of soft biological tissues is estimated to range from 1 to 200 kPa [65,66].

According to Figure 5, the values of $E_{ref}$ obtained during the mechanical characterization of the agar-based soft tissue phantoms with DMA are consistent with the results reported elsewhere with other agar hydrogels and measurement techniques such as magnetic resonance elastography (MRE) [59], DMA [59], quasi-static compression test [60] and rheological tests [61]. Agar hydrogels are usually prepared with a minimum variation in $c_{ag}$ of 0.5%, except for Mishra et al. [61] where the authors considered 4 agar hydrogel samples with $c_{ag}$ ∈ [0.25%, 0.3%, 0.4%, 0.6%]. Another originality of the present study is to present the first collection of agar hydrogels with a small concentration increment (0.1%) over a large interval $c_{ag}$ ∈ [1.5%; 5.4%]. Agar hydrogels have nonlinear stress–strain behaviors [67] and are described by viscoelastic [60] and hyperelastic constitutive laws [68], which should be taken into account in future studies, since the agar hydrogel behavior is influenced by preloading conditions [60,67].

### 4.2. Data Analysis

The correlation matrix and the PCA revealed that the positions of the different peaks $t_{i}$ provided redundant information. Thus, estimating some subsequent $t_{i}$ (e.g., $t_{3}$, $t_{4}$ and $t_{5}$) in addition to our current indicator Δt (i.e., $t_{2}$) could reduce the measurement uncertainties inherent to certain force signal characteristics and consequently, the mechanical properties of soft tissue. Although the findings were not as clear, there could also be redundancy of information between neighboring peaks such as between the values $l_{i-1}$, $l_{i}$ and $l_{i+1}$, or between the values $F_{i-1}$, $F_{i}$ and $F_{i+1}$. This observation could be useful for setting up other indicators aiming at assessing other mechanical properties, such as viscosity and nonlinearity of the material [29]. Additionally, reducing noise in the IBAM set-up could improve visualization of correlations.

The PCA also revealed that approximately 85% of the information from the dataset could be captured by only three variables. It could be interesting to consider the implementation of PCA in the context of predictive ML models, which allows the reduction of the volume of data, thereby accelerating the training process. Furthermore, the projection of the dataset onto the two principal components plane revealed a significant correlation between the $t_{i}$ features and Young’s modulus $E_{ref}$. The other features did not demonstrate any specific relationship to soft tissue phantom stiffness. As a result, the association of Δt (i.e., $t_{2}$) with soft tissue stiffness is the most suitable among the basic characteristics of the force signal ($t_{i}$, $F_{i}$ and $l_{i}$), apart from the other $t_{i}$.

### 4.3. Prediction Models

The comparison of RMSPE prediction errors suggest that AI-based models outperform our current approach based on Δt to identify Young’s modulus $E_{ref}$ of soft tissue phantoms based on the force signal of IBAM. Among the classical ML models, decision trees and stacking ensemble approaches proved to be the most efficient, with a significant reduction of RMSPE up to 79% compared to the IBAM reference. Similarly, the various CNNs demonstrated an improvement in RMSPE, with an average reduction of 50%, compared to the IBAM reference. However, given the attractiveness of DL approaches and the complete and non-simplified force signal data used as input to the models, it would have been expected to obtain the best performance with CNNs. Training and enhancing the performance of CNNs is more challenging due to the complexity of their structures and the large volumes of data involved. For instance, the performance of the different CNNs (1D-CNN and 2D-CNN) appeared to be equivalent. The main difference was in the training time: around 10 min for 1D-CNN versus 3 to 6 h for 2D-CNN. Despite the fact that images provide more data-rich representations [69], using a 1D-CNN enables the reduction of training times and facilitates the adjustment of model structure and hyperparameters. According to Shahid et al. [69], an increase in data volume is necessary for 2D-CNN models to perform better than 1D-CNN models because 2D operations are more computationally demanding.

Using model inspection techniques, the contribution of different peak characteristics (position, $t_{i}$; amplitude, $F_{i}$; and half-width, $l_{i}$) of IBAM force signals to the predicted Young’s modulus $E_{pred}$ could be investigated in the case of ML models only. Except for KNN, the various models relied almost exclusively on the $t_{i}$ features. In the decision tree models, $t_{2}$ was the primary feature, and the only contributive feature, in XGBoost and Random Forest models. In the same way as in data analysis, this result confirms the effectiveness of the Δt indicator for characterizing the stiffness of soft tissue phantoms.

### 4.4. Limitations and Perspectives

There are several limitations associated with the present study. First, only the dynamic Young’s modulus of the soft tissue phantoms was mechanically characterized using a custom vibration set-up. Despite this simple method, it would be interesting to repeat the study and determine Young’s modulus using more conventional approaches. For instance, a universal testing machine could be used to perform standardized materials science techniques, such as DMA, compression or tensile testing. The more conventional approaches would also enable more detailed characterization of the sample’s mechanical properties via viscosity or nonlinearity measurements.

Second, a pre-processing of the raw force signals obtained with IBAM was required for the classical ML models to determine their input features (e.g., interpolation of the first five peaks with $t_{i}$, $F_{i}$ and $l_{i}$). This pre-processing task required prior knowledge of the data and significant preparation time to select features, which could be a limiting factor for very large datasets [34]. However, it is common practice in CNN that feature extraction and output prediction are performed jointly to ensure that models capture information holistically [34]. Compared to ML, this approach allows to recognize complex patterns or relationships within the overall information contained in data [34]. As previously mentioned, these DL approaches are more costly in terms of computation and training data. The transfer learning techniques could be an effective solution for reducing the computational costs, especially for model training.

Third, the predictive performance of the prediction models is limited due to the use of only one group of samples, despite the large number of samples with different stiffness. Therefore, repeating this experiment with other soft tissue phantoms could improve the robustness of the AI-based models. It may be useful to modify the stiffness distribution and also the materials of the samples (e.g., gelatin hydrogels, polyvinyl alcohol gels, silicones, polyurethane gels [70]).

Fourth, the present study must be carried out with biological soft tissues such as the skin, which is much more complex, given its multilayer structure and its nonlinear, viscous, and anisotropic mechanical behavior [71,72]. Due to their interdependence, all skin mechanical and microstructural properties vary in parallel, so that it is impossible to isolate the effect of each property on the IBAM response. For this reason, combining *in silico* approaches with AI-based predictive models could be an interesting perspective to understand the complex behavior of biological soft tissues. A large dataset could be generated by precisely varying the parameters of an analytical [73] or numerical model [74] (e.g., FE model) of the mechanical characterization technique and of the soft tissues. These data could be used to train an AI-based model for identifying the modified mechanical properties of soft tissue. Finally, if this approach is to be used *in vivo*, a significant proportion of IBAM data collected on human soft tissues will have to be included in the training dataset of AI-based predictive models. However, the mechanical behavior of biological soft tissues can fluctuate greatly between individuals due to physiological (e.g., ethnicity, age, hormonal factors, pathologies, etc.) and environmental factors (e.g., lifestyle, UV exposure, temperature, moisture, etc.) [72,75]. It will be crucial to ensure the representativeness and distribution of the training dataset, especially human measurement data, with respect to the target patient groups [76,77]. In the case of population-specific training datasets, AI-based models may not generalize well, which can cause significant performance disparities between individuals.

The IBAM is a surface mechanical characterization device under development, which explains why the current and previous studies [28,29,30] focused on discriminating variations in the stiffness of soft tissue phantoms. However, superficial soft tissues such as cutaneous tissues are not homogeneous, isotropic, elastic materials, but exhibit complex mechanical characteristics (e.g., multilayered, anisotropic, viscoelastic, etc.) [71,72]. Furthermore, the mechanical behavior of the skin is not only subject to inter-individual and pathological variations, but also to intra-individual differences related to anatomical location. Working with soft tissue phantoms has the advantage of obtaining standardized materials. However, some experimental challenges are still associated with soft tissue phantoms (e.g., low reproducibility and difficulty in controlling their mechanical properties [63,64]). *In silico* modeling of IBAM could be an effective solution to improve our understanding of the underlying phenomena.

For this reason, preliminary basic models (a 1D analytical model, an FE model) have been developed to reproduce the configurations of early *in vitro* studies [31]. Modeling could enable an inverse method to estimate certain mechanical properties (e.g., Young’s modulus of skin) in complex tissues from IBAM force signals.

IBAM measurements can be influenced by underlying tissues when characterizing superficial soft tissues. The force signal reflects the average mechanical behavior of the tissues within the volume of interest (VOI) of the IBAM measurement. Bouffandeau et al. [29] demonstrated that this VOI is equivalent to a hemisphere with a radius approximately equal to the diameter of the punch. Therefore, adjusting the diameter of the bottom of the punch can reduce the influence of underlying tissues on the force signal.

At this stage of development, IBAM can only be used for comparative measurements performed at the same site, such as before and after treatment. Furthermore, skin from animal models should be used to validate IBAM in a situation closer to the clinical configuration. Despite the differences in mechanical properties between animal and human tissue, measurements in animal models could provide proof of concept for the applicability of IBAM with regard to various physiological or pathological mechanisms. For example, preliminary *ex vivo* experiments were conducted with murine skin models to assess differences between healthy areas and areas affected by cutaneous neurofibromas [32]. Other *in vivo* measurements were also performed to monitor changes due to wound healing following loss of substance and intradermal hyaluronic acid injection [33]. However, murine skin exhibits considerably less variability in mechanical properties than human skin. The controlled laboratory conditions and shorter lifespan of the animals likely cause this difference by limiting the development of tissue heterogeneity due to lifestyle factors over time. Therefore, these findings will need to be confirmed by performing measurements in human skin using an advanced version of the IBAM (i.e., a handheld, automatic and standardized device). In light of the pronounced intra-individual variability of human skin biomechanics, estimating Young’s modulus requires careful consideration to minimize variability induced by i) the measurement method (e.g., tension, compression, shear, indentation), ii) the loading direction, due to the intrinsic anisotropy of skin, and iii) the scale (macroscopic vs. microscopic). The latter aspect is critical because skin exhibits multiscale properties and may be affected by underlying tissues (e.g., muscle, adipose tissue).

Finally, integrating IBAM into an objective, non-invasive and user-friendly device could create a valuable decision support system for the mechanical characterization of skin. It could enhance diagnosis and treatment evaluation in multiple medical fields, such as dermatology and plastic surgery. Additionally, skin characterization could be of great interest to the cosmetics industry for assessing product efficacy. The accessibility of the device could also be used to provide customer-specific product recommendations. Rather than replacing elastographic techniques for the mechanical characterization of deep organs, the proposed AI-IBAM framework is intended to provide a user-friendly, low-cost, non-invasive and objective solution for the real-time measurement and long-term monitoring of superficial soft tissues in dermatology and cosmetic applications.

## 5. Conclusions

This paper investigated the application of AI-based approaches to our mechanical characterization method, IBAM, to improve the processing of data with respect to the mechanical properties of soft tissue using a collection of 40 agar-based phantoms. Among the various characteristics of the force signal ($t_{i}$, $F_{i}$ and $l_{i}$), features $t_{i}$ were shown to be highly correlated with each other, as well as consistent with the variation in Young’s modulus $E_{ref}$ of the soft tissue phantoms. Except for the linear model, all AI-based models predicted $E_{pred}$ with lower RMSPE than the standard IBAM approach with indicator Δt (${RMSPE}_{\Delta t}$ = 5.84%). The values of RMSPE ranged from 2% to 3% for CNN, and reached 1% to 2% for decision trees and ensemble stacking. An inspection technique revealed that, in this context, the reasoning behind classical ML models was predominantly based on the $t_{i}$ features. Therefore, besides the other $t_{i}$ features, the current indicator Δt (i.e., $t_{2}$) of IBAM appears to be the only basic characteristic of the force signal associated with $E_{ref}$. Eventually, AI-based prediction models could be improved and extended to other mechanical properties such as viscosity, through various methods including increased training data, transfer learning and combination with *in silico* approaches.

## Figures and Tables

**Table 1:** Comparison of root mean square percentage errors RMSPE between Young’s modulus prediction value $E_{pred}$ and reference value $E_{ref}$ for different machine learning and deep learning regression models, compared to the model using the Δt indicator (reference error).

	Method	RMSPE
IBAM	Indicator Δt—(*Reference*)	5.84%
Machine learning	Linear Regression Model	30.43%
	K Nearest Neighbors	2.99%
	Support Vector Machine	2.55%
	Gaussian Process	3.40%
	CatBoost	1.95%
	XGBoost	1.44%
	Random Forest	1.53%
	Ensemble Stacking	1.22%
Deep learning	1D-CNN *(force vector)*	2.22%
	2D-CNN *(temporal force image)*	3.26%
	2D-CNN *(wavelet transform image)*	3.23%

## Data Availability

The data and models are stored in the MSME laboratory.

## Abbreviations

The following abbreviations are used in this manuscript:

AI	Artificial Intelligence
ANN	Artificial Neural Networks
CNN	Convolutional Neural Networks
DL	Deep Learning
DMA	Dynamic Mechanical Analysis
FE	Finite Element
IBAM	Impact-Based Analysis Method
KNN	K-Nearest Neighbors
ML	Machine Learning
MRE	Magnetic Resonance Elastography
OCE	Optical Coherence Elastography
PCA	Principal Component Analysis
RMSPE	Root Mean Squared Percent Error
SVM	Support Vector Machine

## Appendix A

**Table A1:** List of hyperparameters implemented in GridSearch for machine learning models: k-nearest neighbors (KNN), support vector machine (SVM), gaussian process and decision trees (CatBoost, XGBoost and Random Forest). Options in bold corresponded to optimal values for hyperparameters.

Model	Hyperparameters	Options
k-Nearest Neighbors	Number of neighbors	1; 2; 3; 4; 5; 6; 7; **8**; 9; 10; 11; 12; 13; 14; 15
	Weight function	Uniform; **Distance**
	Algorithm	**Auto**; BallTree; KDTree
	Distance metric	Minkowski; Euclidean; **Manhattan**
Support Vector Machine	Kernel type	Linear; Poly; **Radial Basis Function**; Sigmoid
	Kernel coefficient	0.01; **0.05**; 0.1; 0.5; 1
	Regularization parameter	1; 10; 100; 500; 1000; 1500; **2000**
Gaussian process	Covariance function	**Radial Basis Function**; DotProduct
	Length scale	0.1; **10**
	Alpha	0.001; **0.01**
CatBoost	Number of trees	100; 200; 400; 600; 800; 1000; 1200; 1400; **1600**
	Learning rate	0.01; **0.05**; 0.1; 0.2
	Depth of trees	4; 5; **6**; 7; 8; 9; 10
	L2 regularization	1; **3**; 5; 7; 9
XGBoost	Number of trees	200; 400; 600; 800; 1000; 1200; 1400; **1600**
	Learning rate	0.005; **0.01**; 0.05; 0.1
	Max tree depth	5; 6; 7; **8**; 9; 10
	Subsample	0.3; **0.5**; 0.7; 1
	L2 regularization	**0.5**; 1; 3; 5
Random Forest	Number of trees	100; **200**; 400; 600; 800; 1000; 1200
	Max tree depth	3; 4; 5; 6; 7; 8; 9; **10**
	Min samples split	**2**; 5; 10
	Min samples leaf	**1**; 2; 4; 6
	Criterion	**Squared error**
	Bootstrap	**True**; False
